# Blood Pressure and the Capacity-Load Model in 8-Year-Old Children from Nepal: Testing the Contributions of Kidney Size and Intergenerational Effects

**DOI:** 10.1002/ajhb.22829

**Published:** 2016-02-05

**Authors:** Jonathan C.K. Wells, Delan Devakumar, Carlos S. Grijalva-Eternod, Dharma S. Manandhar, Anthony Costello, David Osrin

**Affiliations:** 1 Childhood Nutrition Research Centre, UCL Institute of Child Health, London, United Kingdom; 2 UCL Institute for Global Health, London, United Kingdom; 3 Mother and Infant Research Activities, Kathmandu, Nepal

## Abstract

**Objectives:**

Growth patterns in early life are increasingly linked with subsequent cardio-metabolic risk, but the underlying mechanisms require elucidation. We have developed a theoretical model of blood pressure, treating it as a function of homeostatic metabolic capacity, and antagonistic metabolic load. We sought to differentiate prenatal and postnatal components of metabolic capacity, and to identify intergenerational contributions to offspring capacity and load.

**Methods:**

We followed up at 8 years a cohort of children originally recruited into a randomized trial of maternal micronutrient supplementation in pregnancy. Maternal anthropometry was measured at recruitment. Offspring anthropometry was measured at birth, 2 years and 8 years. Offspring blood pressure, kidney size, and body composition were measured at 8 years. Regression analysis was used to investigate potential associations of maternal phenotype, birth phenotype, and current body composition with kidney size and blood pressure.

**Results:**

Blood pressure was positively associated with body fat, but negatively associated with birth weight and relative leg length. Kidney size was positively associated with birth weight but not with relative leg length. Adjusting for adiposity, blood pressure was independently negatively associated with birth weight, relative leg length, and kidney length. Maternal height and BMI predicted offspring size at birth and at 8 years, but not blood pressure.

**Conclusions:**

Our data provide support for the capacity-load model of blood pressure in Nepalese children. Fetal and postnatal growth and kidney dimensions all contribute to metabolic capacity. Maternal phenotype contributed to offspring capacity and load, but these associations did not propagate to blood pressure.

## Introduction

There is now compelling evidence that growth patterns in early life are associated with subsequent metabolic phenotype (Barker et al., 1989; Bhargava et al., 2004; Eriksson et al., 2006; Hales et al., 1991; Lakshmy et al., 2011; Leon et al., 1996; Lithell et al., 1996). The risk of noncommunicable diseases (NCDs) accumulates from childhood onwards, but is compounded by the effects of poor growth in fetal life and infancy. The greatest risk of chronic disease is, therefore, found in those born small who subsequently become large (Adair and Cole, 2003; Barker et al., 2005; Bavdekar et al., 1999; Eriksson et al., 2006; Fagerberg et al., 2004), although current lifestyle is also influential. Birth weight tends to be lower in south Asian than in European populations, and this may contribute to the high prevalence of chronic diseases in populations from the Indian subcontinent that are experiencing rapid economic transition (Misra and Khurana, 2009; Yajnik et al., 2003).

To shed more light on these associations between early growth and later risk, we have built on the thrifty phenotype hypothesis of Hales and Barker (1992). They hypothesized that nutritional insufficiency *in utero* induced the fetus to sacrifice growth of some organs (e.g., pancreatic beta-cell mass, muscle mass, liver, and kidney mass) in order to spare the brain (Hales and Barker, 1992; Latini et al., 2004). Such reallocation of energy between organs and tissues would favor short-term survival, but at the cost of reduced tolerance of “affluent lifestyle” in later life. However, while this approach emphasized the detrimental effects of low birth weight, it did not explain why inverse associations between birth weight and later disease risk are apparent across the entire range of birth weight (e.g., Rich-Edwards et al., 1997), and hence apply also to those with adequate fetal growth. Others have clarified that postnatal weight gain at any age is positively associated with later blood pressure, mediated by increases in tissue masses (Adair et al., 2009; Leunissen et al., 2012).

To give a clearer physiological basis to the thrifty phenotype, we developed a “capacity-load” conceptual model that treats chronic disease risk as the product of two interacting traits (Wells, 2009, 2011). This approach assumes that the capacity to maintain homeostasis is promoted by growth during early life, as demonstrated, for example, by positive associations between birth weight and various metabolic traits (Wells, 2011). “Metabolic capacity,” a generic term for such homeostatic ability, is thus assumed broadly to increase in proportion to birth weight (although macrosomic infants may deviate from this broad pattern), and to protect against chronic disease risk in the long term.

Nevertheless, for some traits, critical windows for the acquisition of metabolic capacity may extend beyond fetal life into infancy (Wells, 2009, 2014). Relative leg length may, therefore, act as an additional proxy for certain components of metabolic capacity, as it indexes early postnatal growth independently of fetal weight gain (Bogin and Baker, 2012; Gunnell et al., 1998, 1999; Pomeroy et al., 2014). A recent study from Peru found that high-stress environments were associated with greater reductions in lower leg length than in other components of linear growth (Pomeroy et al., 2012), supporting the notion that leg length is a useful marker of early growth. However, as yet there is little information on the variable duration of developmental plasticity in traits relevant to metabolic capacity.

We then assume that NCD risk increases in proportion to the accumulation of “metabolic load,” which challenges the capacity for homeostasis (Wells, 2009, 2011). Given the health risks of obesity, we treat adiposity as a key component of metabolic load. However, other components of body size (height, lean mass) may also elevate load, as may components of lifestyle such as lipogenic or high-salt diets and sedentary behavior (Andersen et al., 2006; Ford et al., 2005; Lee et al., 2012; McKeown et al., 2004; Willett et al., 2002). All of these challenge the maintenance of homeostasis at the level of cells or physiological systems.

We recently tested the capacity-load model in relation to blood pressure in a large sample of UK children aged 9 years (Grijalva-Eternod et al., 2013). As nephron number scales positively with birth weight, and cannot increase in postnatal life, birth weight may act as a reliable marker of this component of metabolic capacity (Luyckx et al., 2013; Manalich et al., 2000). Increased body weight is predicted to act as a metabolic load through a variety of pathways, relating to both lean and fat mass (Belin de Chantemele et al., 2011; Sobotka et al., 2011; Weder and Shork, 1994). Consistent with the predictions, height, lean mass, and adiposity were all independently associated positively with blood pressure, whereas birth size was negatively associated (Grijalva-Eternod et al., 2013). The strongest component of load was adiposity. We obtained similar findings in a second test on adults from southern Italy, whose birth weights were not available, but where we could take into account different components of height: adiposity, lean mass, and trunk height were associated positively with blood pressure (although variably so between the sexes), whereas greater relative leg length was associated with lower blood pressure (Montagnese et al., 2014). It remains unclear what component of metabolic capacity might be benefitted by longer legs, and as yet, these studies have not measured metabolic capacity directly through, for example, kidney dimensions or functional properties.

During early life, the offspring is exposed not directly to the external environment, but to maternal physiology (Wells, 2003, 2007). This means that any adaptive element of offspring growth strategy occurs in relation to maternal phenotype, which can be regarded as “maternal capital” for investment in the offspring (Wells, 2010). The importance of maternal capital for the offspring’s development lies in the fact that critical windows of offspring plasticity match closely with the duration of exposure to maternal capital (Wells, 2014).

Maternal capital has been hypothesized to buffer the fetus from short-term ecological variability (Kuzawa, 2005; Wells, 2003, 2010), which means that the offspring is exposed to more stable components of maternal phenotype such as body size and body composition. Thus, it is important to consider how maternal capital may be associated with metabolic capacity and load in the offspring. Previous studies have shown that offspring birth weight is correlated with maternal birth weight and grand-maternal height (Emanuel et al., 1992; Hyppönen et al., 2004), indicating trans-generational effects that may incorporate both genetic and nongenetic mechanisms. Maternal size similarly appears an important predictor of patterns of postnatal growth (Azcorra et al., 2013; Varela-Silva et al., 2009).

To address these issues in more detail, we tested the capacity-load model of blood pressure in a sample of Nepalese children aged 8 years, whose mothers had been recruited during early pregnancy into a randomized trial of micronutrient supplementation (Osrin et al., 2005; Vaidya et al., 2008). This trial showed a modest but significant 77 g increase in birth weight in those whose mothers received the composite micronutrient supplement, compared with those whose mothers received only iron and folic acid (Osrin et al., 2005). Data on the mothers were available at the time of recruitment, while offspring phenotype was assessed at birth and 2 years. We conducted a further follow-up at 8 years of age (Devakumar et al., 2014), measuring offspring growth, body composition and blood pressure. To incorporate more direct measures of metabolic capacity in our model, we also measured dimensions of the kidneys by ultrasound.

**Fig. 1:**
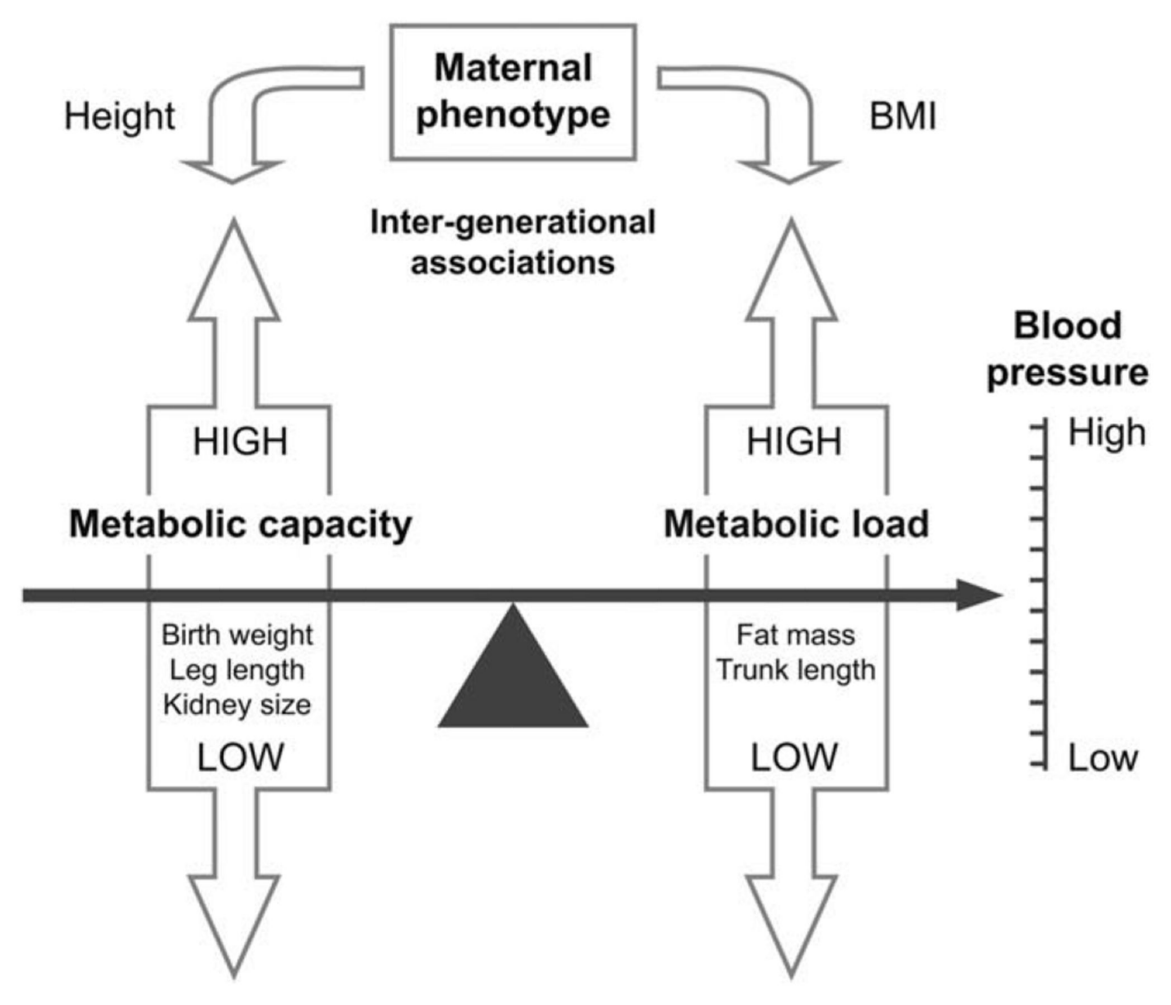
Conceptual diagram, illustrating the expected complementary contributions of metabolic capacity and metabolic load to blood pressure, and their potential to be shaped by maternal phenotype.

We predicted that height and fat and lean mass at 8 years would represent components of metabolic load, and would correlate positively with blood pressure. We then predicted that birth weight and relative leg length would index components of metabolic capacity, and would correlate negatively with blood pressure. We predicted that these associations would be mediated by kidney dimensions, and that maternal capital (height and body mass index [BMI]) would correlate positively with offspring metabolic capacity, and negatively with offspring blood pressure (Fig. 1).

## Methods

### Original trial

The study was conducted in Dhanusha district, in the lowland Central Terai region of Nepal. The original trial has been described in detail elsewhere (Osrin et al., 2005). It is registered as an International Standard Randomised Controlled Trial, number ISRCTN88625934.

Briefly, 1,200 women attending Janakpur Zonal Hospital for antenatal care were randomly allocated to receive either the UNIMMAP micronutrient supplement (vitamins A, B1, B2, B6, B12, C, D, E, and niacin, along with folic acid, iron, zinc, copper, selenium, and iodine; Danish Pharmaceutical Industries, Denmark) or a control supplement of iron and folic acid. The supplements were taken daily from between 12 and 20 weeks gestation until delivery. Exclusion criteria included multiple pregnancies, fetal abnormalities on obstetric ultrasound, and maternal illness that could compromise the outcome of the pregnancy.

A total of 1,069 mothers and infants completed the trial and were assessed at birth. Maternal anthropometry was measured at the time of recruitment into the trial. At 2 years, a follow-up was conducted in which weight and height were among the outcomes assessed.

The original trial was approved by the Nepal Health Research Council and by the ethics committee of the Institute of Child Health and Great Ormond Street Hospital for Children, UK, and was undertaken in collaboration with the Nepal Government Ministry of Health. The 8-year follow-up was approved by the same institutions. Verbal and written informed consent was taken from parents or guardians in their local language.

### 8-Year follow-up

Every attempt was made to find the children from the original trial using location data from previous follow-up. The follow-up included 14 children who had moved to Kathmandu or the town of Hetauda. The study was powered at 81% to detect a difference of 0.2 standard deviation scores between the allocation groups, with a sample size of 400 in each group, at 5% significance level. Thus, the intention was to remeasure a minimum of 800 of the original sample.

Data were collected on birth order of the child and of the mother. Anthropometry measurements were conducted in accordance with UCL Institute of Child Health guidelines, adapted from Lohman et al. (1988) and the WHO Multi-Centre Growth Reference Study Group (2006). Duplicate measures of standing height were measured barefoot and with the head in the Frankfort plane, using a Leicester stadiometer (Invicta Plastics, UK), accurate to 0.1 cm. Sitting height was measured using a custom-made stool, with the base of the spine touching the stadiometer and head in the Frankfort plane. Leg length was calculated as the difference between sitting height and height, and relative leg length was calculated as (leg length/height).

Weight and body composition were measured using a Tanita BC-418 scale (Tanita Corp, Japan) scale accurate to 0.1 kg. Children wore standardized clothing weighing 200 g. Raw impedance was converted to body composition values using an isotope-calibration study conducted on a subsample of 100 children (Devakumar et al., 2015). BMI was calculated as weight/height^2^, and weight, height, and BMI z-scores were calculated using WHO reference data (WHO Multi-Centre Growth Reference Study Group, 2006). Skinfold thicknesses at the biceps, triceps, subscapular, and suprailiac sites were measured using a Harpenden calliper (Assist Creative Resource, Wrexham, UK), accurate to 0.2 mm.

Blood pressure was measured with an Omron M6 electronic blood pressure monitor (Omron Healthcare, Japan) with a pediatric or adult cuff as required. Measurements followed Great Ormond Street Hospital for Children (2010) guidelines. Blood pressure was recorded after the child had been seated for at least 1 min with legs uncrossed. Two readings were taken 1 min apart, with the cuff deflated fully between them. The lowest value was recorded.

Ultrasound measurements of kidney size were taken by a local clinician trained in ultrasonography using Aloka SDD-500 instrumentation with a 2–8 MHz convex probe (Aloka Co., Japan), accurate to 1 mm. The maximum renal length and antero-posterior diameter were recorded, ensuring that the sinus and parenchyma were visualized using predefined landmarks. Kidney area for each side was calculated as (length * antero-posterior diameter * pi/2), and the average area was calculated. Technical error of the mean (TEM) values were calculated from repeat measurements in a subsample of approximately 5% of the total. TEM values were 0.21 cm (2.6%) and 0.16 cm (1.9%) for the right and left kidney lengths, respectively, and 0.14 cm (4.7%) and 0.19 cm (5.7%) for the right and left kidney antero-posterior diameters. In each case, there was no evidence of systematic bias between first and second measurements.

Children in whom an illness was suspected from our investigations were referred to a local pediatrician for more detailed investigation if required, for which the costs were covered. A large proportion of children who were stunted (height for age ≤ −2 SD) or of low BMI were referred to a local nutrition center. All children were also given a T-shirt, refreshments, and a voucher to be seen by a local pediatrician, external to the research team, with the costs of minor acute treatments covered.

### Conceptual model and statistical analysis

To operationalize the capacity-load model, we initially tested height, lean mass, and adiposity indicators as components of metabolic load, and birth weight as the primary index of metabolic capacity. Correlation and regression analyses were used to test these associations between early growth and later body composition. We also stratified the capacity-load analysis by trial group, to test whether the association between birth weight and later blood pressure was similar in those whose mothers received composite micronutrient supplementation, and those whose mothers received the basic iron and folic acid supplement.

We then introduced relative leg length (or sitting height and leg length separately) and kidney dimensions into the model as potential independent components of metabolic capacity, or as mediating factors. The two kidney measurements were not sufficiently strongly correlated to cause problems with collinearity.

Associations between maternal and offspring size and body composition were tested using correlation analysis. We then introduced maternal size into the capacity-load regression models, to see if direct trans-generational influences on blood pressure could be detected.

Fat mass and sum of skinfolds were right-skewed, as was maternal BMI. These variables were log-transformed prior to analysis. Left and right kidney measurements were averaged. Conditional growth for height and BMI was calculated in the following way. Size at the second time point was regressed on the same variable at a prior time point and the regression residual taken. Using this approach, we obtained values for height at 2 years conditional on length at birth, and did likewise for BMI at 2 years conditional on birth weight (we could not generate BMI *z*-scores at birth due to the lack of reference data). We then obtained similar outcomes by regressing 8-year values on 2-year values, to give conditional height and conditional BMI at 8 years. In this way, we were able to explore associations of early versus late growth in height and BMI with later outcomes.

## Results

A total of 841 children, 70% of the original sample, participated in the 8-year follow-up. Those who could not be located had higher maternal weight and BMI than those who participated, but no difference in birth weight, birth length, maternal age, or height. In the original trial, birth weight was greater in the intervention group than the control group (77 g; 95% CI 24, 130) (Osrin et al., 2005), but there was no difference between the two trial groups in any of the outcomes at 8 years (Devakumar et al., 2014), or in maternal characteristics. For this reason, and because it was not significant in statistical models, allocation group was not included in the main analyses described below.

Amongst those participating at 8 years, relative to WHO reference data, mean (SD) z-scores were −2.0 (1.0) for weight, −1.5 (0.9) for height, and −1.6 (1.0) for BMI. Table 1 provides data aggregated by sex for size at birth, size, and body composition at 8 years, and maternal size. Boys were significantly heavier (94.2 g; 95% CI 37.0, 151.5) and longer (0.2 cm; 95% CI 0.08, 0.87) at birth, but there were no sex differences in terms of maternal height or BMI. At 8 years, boys were significantly heavier and taller, and had greater BMI, lean mass, relative leg length, and kidney AP diameter, but significantly lower fat mass and sum of skinfolds. There was no significant difference in size at birth, or in any outcome at 8 years, between first-borns and later-borns (data not shown).

Maternal height correlated with birth weight (r = 0.22, *P* < 0.0001) and birth length (r = 0.14, *P* < 0.0001), while maternal BMI also correlated with birth weight (*r* = 0.15, *P*< 0.0001), but only very weakly with birth length (r = 0.04, *P* = 0.053).

### Offspring growth and body composition at 8 years

**Fig. 2:**
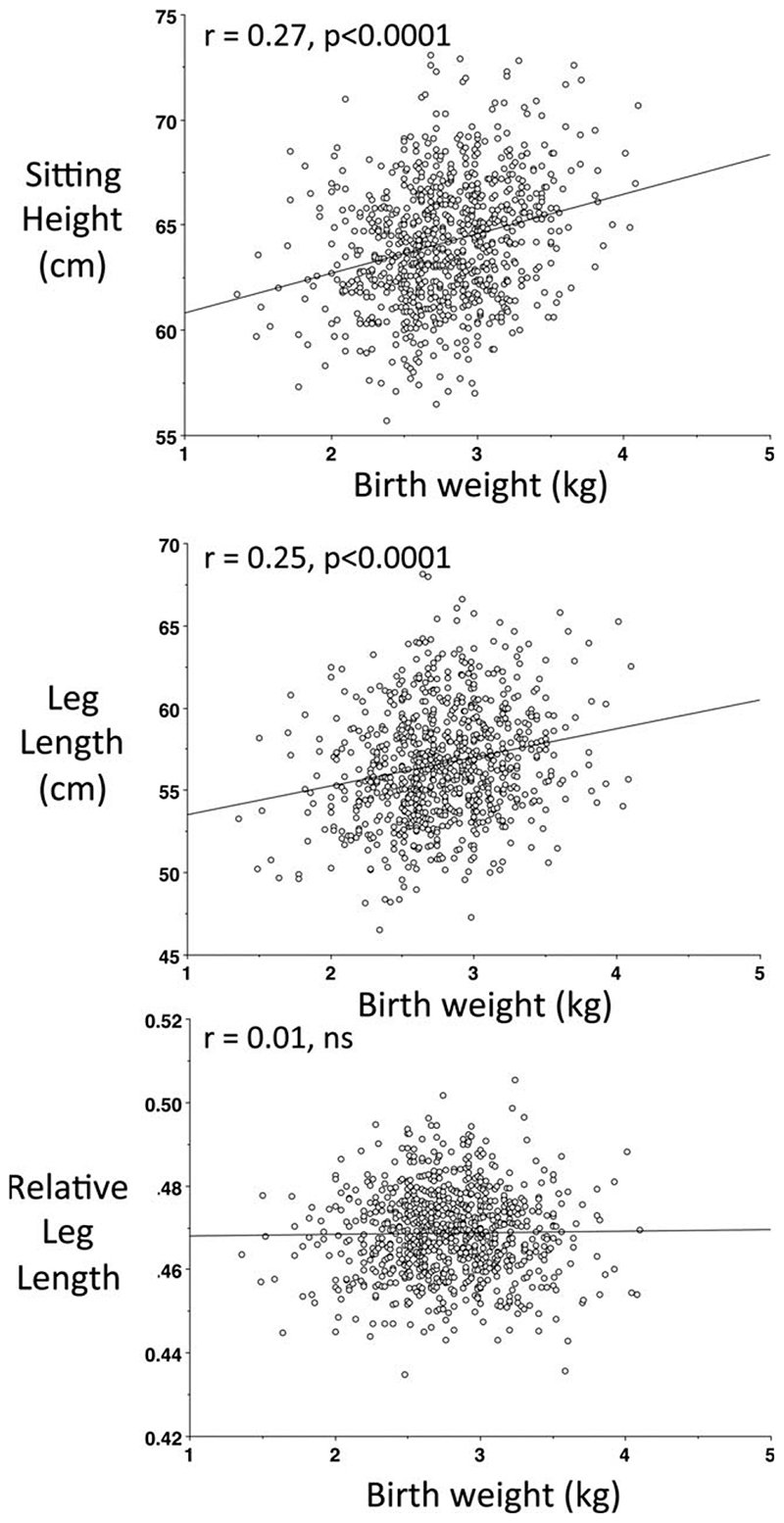
Associations of birth weight with (a) sitting height, (b) leg length, and (c) relative leg length.

**Fig. 3:**
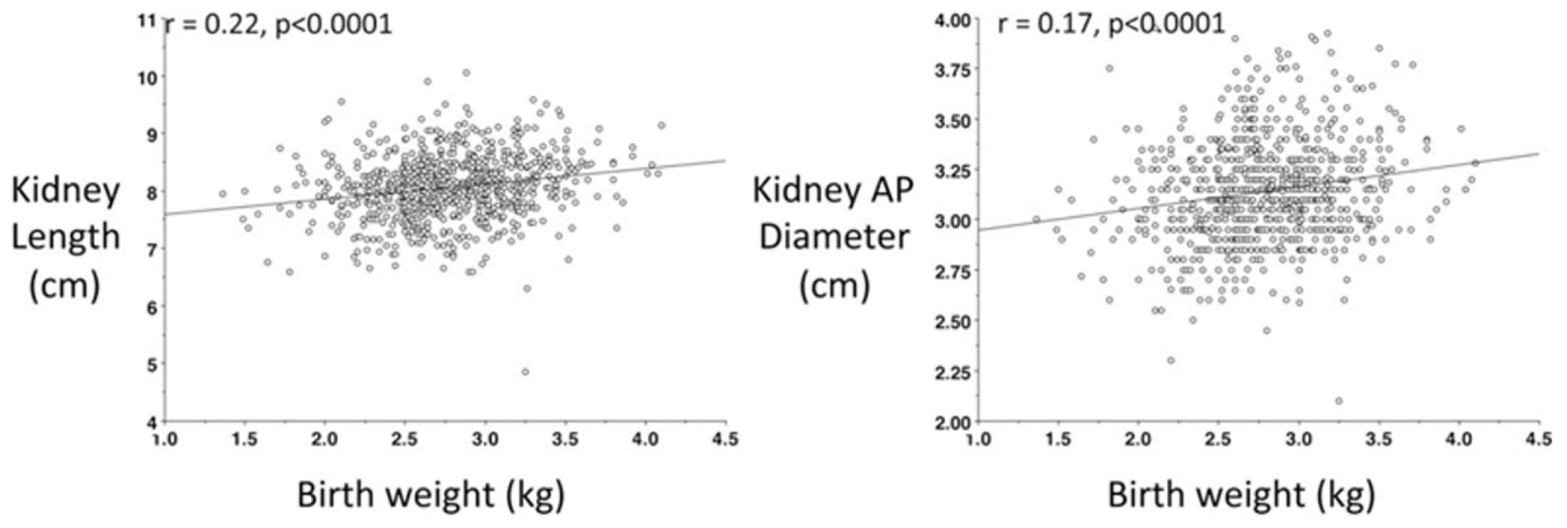
Association of birth weight with (a) length and (b) antero-posterior diameter of the kidney (averaged for the two kidneys).

Correlations between size at birth and phenotype at 8 years are shown in Table 2. Birth weight and birth length were both positively associated with sitting height and leg length, indicating that fetal linear growth is positively associated with both the main components of childhood height. However, there was no association between size at birth and relative leg length (Fig. 2). Birth weight correlated significantly with weight, height, BMI, lean mass, Ln fat mass, Ln sum skinfolds, and kidney dimensions, but not with blood pressure. Results for birth length were very similar, although it correlated with relative leg length, but not with Ln sum skinfolds. The correlations for the kidney measurements on the two sides of the body were 0.77 (*P* < 0.0001) for length and 0.38 for the AP diameter (*P* < 0.0001). Figure 3 illustrates the association of birth weight with (a) kidney length and (b) kidney AP diameter. Correlations of kidney dimensions were marginally stronger with birth weight than with birth length.

Table 3 describes the correlations of somatic traits and kidney dimensions at 8 years. The correlation between kidney length and AP diameter was 0.41 (*P* < 0.0001). Kidney length correlated significantly and directly with weight, height, BMI, lean mass, Ln fat mass, and Ln sum of skinfolds. Kidney AP diameter correlated with the same traits, although generally less strongly, and also with systolic blood pressure. When entered together into a regression model, both kidney dimensions were associated with Ln fat mass, with the coefficient for AP diameter (*B* = 0.239, SE = 0.063) being stronger than that for kidney length (*B* = 0.168, SE = 0.029). However, when height was the outcome, the coefficient for kidney length (*B* = 5.15, SE = 0.36) was greater than that for AP diameter (*B* = 2.93, SE = 0.79).

### The capacity-load model

Tests of the capacity-load model are given in Table 4. Adjusting for the positive association of fat mass with blood pressure, birth weight, and relative leg length were independently negatively associated with blood pressure. Similar results were obtained if Ln sum of skinfolds replaced Ln fat in the model, although coefficients were generally reduced, and relative leg length did not achieve significance for diastolic blood pressure. Neither gender nor birth length had an effect in these models or in the more complex ones described below. Introducing the two components of height separately into the regression model, leg length was inversely associated with blood pressure, whereas sitting height showed either no association or a positive association with blood pressure.

Stratifying the capacity-load analysis by trial allocation group, the inverse association between birth weight and later systolic blood pressure was significant in the multiple micronutrient group (P = 0.048), but not in the group whose mothers received only iron and folic acid (P = 0.3) (Table 5), though a formal test for a *group-birth weight* interaction was not significant. In contrast, the two groups showed little difference in their association of relative leg length and systolic blood pressure.

Subsequent models tested birth weight and relative leg length simultaneously, while also including kidney dimensions (Table 6). For systolic blood pressure, adjusting for the direct effect of Ln fat mass, each of birth weight, relative leg length, and kidney length were independently negatively associated with blood pressure, whereas kidney AP was positively associated. These findings were broadly similar if Ln fat mass was replaced by Ln sum of skinfolds, but birth weight was only marginally significant in this model. For diastolic blood pressure, adjusting for the direct association of Ln fat mass, kidney length was negatively associated with blood pressure, while birth weight and relative leg length showed marginal negative associations. When Ln fat mass was replaced by Ln sum of skinfolds, only birth weight was significant in the model, with a negative association with blood pressure.

We also developed regression models for blood pressure that included both birth length and leg length (data not shown). Birth length was not significant in these models, and when it was removed, the coefficient for leg length changed negligibly.

We analyzed conditional growth, to test associations of height and BMI increases during early life (birth, 2 years) or during later childhood (2–8 years) with kidney dimensions at 8 years. Conditional height at 2 years was associated more strongly with kidney length (*r* = 0.33, *P* < 0.0001) than with kidney AP (*r* = 0.24, *P* < 0.001). Conditional height at 8 years was again more strongly associated with kidney length (*r* = 0.29, *P* < 0.0001) than with kidney AP (*r* = 0.13, *P* < 0.001). Conditional BMI at 2 years was weakly associated with kidney length (*r* = 0.08, *P* = 0.025) and kidney AP (*r* = 0.15, *P* < 0.001), while conditional BMI at 8 years was more strongly associated with kidney length (r = 0.21, *P* < 0.0001) and kidney AP (r = 0.20, *P* < 0.0001).

### Maternal effects

One mother was extremely short, with height ~10 standard deviations below the mean, while her BMI was ~8 standard deviations above the mean. This mother was excluded from the analyses as an extreme outlier, although her offspring was within the normal range for size and body composition and was included in other analyses.

Associations between maternal anthropometry and offspring birth size are shown in Table 2. Maternal height was moderately positively associated with offspring weight, height, and kidney length, but less strongly with Ln sum of skinfolds and relative leg length, and not with offspring blood pressure. Ln maternal BMI was less strongly associated with offspring height and relative leg length, and was not associated with kidney dimensions, but was more strongly associated with offspring BMI and Ln sum of skinfolds. Although maternal height and BMI were associated with components of capacity and load in the offspring, there was no direct association between maternal traits and offspring blood pressure.

## Discussion

This study tested the capacity-load model of blood pressure in 841 children aged 8 years from lowland Nepal. Consistent with the model, both birth weight and relative leg length (treated as markers of metabolic capacity) were negatively associated with blood pressure, whereas adiposity (treated as a marker of metabolic load) was positively associated. As total height and lean mass did not contribute to models of blood pressure, they appeared unimportant components of metabolic load at this age in this population. However, using a more detailed approach, trunk height and leg length showed contrasting associations with blood pressure. Independent of these associations, kidney length was negatively associated with blood pressure, whereas kidney AP diameter was positively associated. Kidney dimensions may, therefore, contribute to variability in blood pressure regulation independently of markers of growth in early life. Finally, maternal height and BMI were associated with components of both metabolic capacity and load in the offspring, but there was no direct association between maternal phenotype and offspring blood pressure.

### Previous studies in South Asian populations

Previous studies of the association between birth weight and later blood pressure in South Asian populations have produced inconsistent findings. In some studies, an inverse association between birth weight and later BP has been reported (Bavdekar et al., 1999; Stewart et al., 2010; Thomas et al., 2012), similar to other populations, whereas in several other studies no such relationship was observed (Kumar et al., 2004; Kumaran et al., 2000; Joglekar et al., 2007). This heterogeneity is poorly understood and the capacity-load model may help elucidate it.

Recent work has identified that South Asian populations have smaller kidneys on average at birth than Europeans, even adjusting for birth weight (Roderick et al., 2016), suggesting that these populations generically have a low metabolic capacity. Our hypothesis is that any association of birth weight with later blood pressure emerges most strongly when adjustment is made for the magnitude of metabolic load at the time when blood pressure is measured. Thus, populations maintaining a healthy lifestyle and low metabolic load may not indicate any adverse effects of diminished metabolic capacity. This scenario would be consistent with the notion that the association between birth weight and blood pressure amplifies with age (Moore et al., 1999), on the assumption that metabolic load also increases with age, as is the case with BMI and, in populations undergoing the nutrition transition, adiposity. Therefore, the capacity-load model merits evaluation in such populations.

### Leg length and birth weight as markers of early growth

Several studies have reported that intra-uterine growth retardation reduces femur length, indicating that shorter legs at subsequent ages might index fetal nutritional constraint (Goetzinger et al., 2012; Todros et al., 1996; Vermeer and Bekker, 2013). In contrast, postnatal growth constraint may disproportionately affect tibia length (Pomeroy et al., 2012). However, although in our sample birth weight and birth length were each positively associated with sitting height and leg length, no association between size at birth and relative leg length was detected, similar to data from previous studies (Bogin and Baker, 2012; Gunnell et al., 1999). A recent study also suggested that relative knee height indexes environmental influences more strongly than genetic influences (Vázquez-Vázquez et al., 2013). Collectively, this evidence justifies the use of relative leg length as a marker of postnatal growth variability, although data on tibia and femur length might have provided additional information about growth variability.

### The capacity-load model

Our findings are broadly consistent with two previous tests of the capacity-load model (Grijalva-Eternod et al., 2013; Montagnese et al., 2014), as well as a similar approach addressing combined effects of birth weight and “unhealthy adult lifestyle” (Li et al., 2015), and suggest that a variety of different body components contribute to variability in blood pressure. However, the overall amount of blood pressure variance explained by these physical traits was very modest, reaching a maximum of 6% in the best model. Relative to WHO reference data, the children were on average short and of low weight and BMI. These characteristics would act to decrease metabolic load, potentially reducing its ability to explain blood pressure variability. In turn, the ability of indices of metabolic capacity to explain blood pressure variability may decline when metabolic load is low. The coefficient of variation for height was ~5%, that for lean mass ~14%, and that for fat mass ~48%. These contrasting magnitudes of variability may help explain why adiposity was the component of load most strongly associated with blood pressure, as may the contrasting associations of leg length versus sitting height with blood pressure, discussed below.

Of particular interest, birth weight and relative leg length manifested as independent components of metabolic capacity. However, when models included both birth length and leg length, birth length was not significant, and when it was removed the coefficient for leg length changed negligibly, suggesting that the magnitude of linear growth achieved specifically in fetal life was not an important predictor of blood pressure in this population. Rather, the magnitude of growth in postnatal life, indexed by leg length or relative leg length, was inversely associated with childhood blood pressure.

Birth weight is known to be strongly associated with nephron number, a trait that is fixed at birth and, therefore, not sensitive to postnatal experience (Manalich et al., 2000). Low nephron number can, therefore, be attributed exclusively to poor growth during fetal life, and relative leg length must, therefore, index other components of metabolic capacity relevant to blood pressure and associated with postnatal growth patterns. It might be assumed that kidney size would track growth of the legs in early life; for example, longer legs might index higher nephron mass. However, our regression models suggested that kidney length and relative leg length contributed independently to blood pressure variability. Other studies have shown that, whereas longer legs are associated with lower blood pressure, longer trunk length is associated with higher blood pressure (Langenberg et al., 2003; Montagnese et al., 2014). The net effect of these contrasting associations is that relative leg length is inversely associated with blood pressure, but we are unable as yet to explain these findings in physiological terms.

The intervention trial provided an opportunity to test whether increasing birth weight does indeed reduce subsequent blood pressure. Consistent with our model, the intervention group was 77 g heavier at birth weight and at 2 years had 2.5 mm Hg lower systolic blood pressure (Vaidya et al., 2008). However, inconsistent with our model, no difference in blood pressure remained evident at 8 years (Devakumar et al., 2014). Interestingly, we observed that the inverse association between birth weight and later blood pressure was stronger, and only significant, in the group whose mothers had received the composite micronutrient supplement during pregnancy, although the magnitude of association between birth weight and blood pressure did not differ significantly between the two trial groups. Thus, we found weak evidence that the benefits of birth weight for later blood pressure were stronger if complemented by maternal micronutrient supplementation. Further follow-up will be required to test in more detail whether the pregnancy intervention generated only a transient effect, or whether a beneficial effect of metabolic capacity will re-emerge later in the life-course when metabolic load is higher.

Another factor contributing to our findings could have been “white coat” effect during the measurement of blood pressure, as the children would not have been familiar with these measurements. Other studies have reported higher vascular reactivity to stressors in adulthood in those born small or after intrauterine growth retardation (Feldt et al., 2008; McCormick Covelli, 2006; Painter et al., 2006). As the physiological toleration of stress may also be considered a component of homeostasis, this could still be considered under the umbrella of a broad capacity-load model, in which greater nutritional investment in early life promotes multiple components of metabolic capacity.

### Kidney length and AP diameter

The contrasting associations of kidney length and kidney AP diameter with blood pressure are intriguing, and it is possible that these two outcomes may reflect different aspects of kidney growth and plasticity. Previous work has found that malnourished children have reduced kidney lengths and lower renal volume during childhood, the main mediating factor being shorter stature (Ece et al., 2007; Gopal and Premalatha, 2014; Morrison and Alleyne, 1976). These reductions indicate lower nephron mass, and although they do not necessarily reduce kidney function during childhood (Gopal and Premalatha, 2014), they may accelerate decline in renal function over the longer term (Brenner and Mackenzie, 1997).

We found that linear growth in both infancy and childhood was associated with kidney length, whereas its association with kidney AP diameter was weaker and declined from infancy to childhood. Conversely, we found that associations of conditional BMI with kidney dimensions increased from infancy to childhood, and were at least as strong for kidney AP as for kidney length. Developmental ablation of kidney mass may elicit glomerular hypertrophy in response. Of particular interest, a previous UK study found that intrauterine growth restriction reduced the AP diameter of the kidney more than its length (Konje et al., 1997). In adults, obesity is associated with glomerulomegaly (Kambham et al., 2001). However, while obese children have longer kidneys (Pantoja Zuzuárregui et al., 2009), it is not yet clear whether they also have kidneys with greater AP diameter.

Overall, our data suggest that having longer kidneys is associated with better blood pressure regulation, whereas having “thicker” kidneys is not. Further work will be required to test these ideas in more detail.

### Influence of maternal capital

Maternal capital has a number of different dimensions which act as proxies for different life-course periods of capital accumulation (Wells, 2010). At birth, maternal height, acting as a proxy for developmental experience, was associated with both weight and length of the offspring, whereas maternal BMI, representing current nutritional status, was associated only with birth weight.

In childhood, maternal height was associated most strongly with offspring height and relative leg length, as well as dimensions of the kidneys. For each additional centimeter of maternal height, offspring kidney length increased by 0.020 cm, and AP diameter by 0.005 cm. Maternal height was weakly associated with offspring fat mass, but not with the sum of skinfolds. In contrast, maternal BMI was not associated with offspring relative leg length or kidney dimensions, but was associated with offspring BMI, Ln fat mass and Ln sum of skinfolds.

Broadly, therefore, maternal completed growth was associated with offspring metabolic capacity, whereas maternal adiposity was associated with offspring adiposity. This suggests that both metabolic capacity and load propagate across generations, but because maternal BMI may be more responsive to short-term environmental stresses the transmission of metabolic capacity is likely to be more stable than the transmission of load. Our data support the maternal capital model (Wells, 2010), showing that greater capital promotes a physiological marker of metabolic capacity in the offspring. However, we were unable to demonstrate direct associations between maternal phenotype and offspring blood pressure, most likely because our childhood indices of capacity and load themselves explained only a modest proportion of variability in blood pressure. A much larger sample size would, therefore, be required to detect the direct influence of maternal effects on offspring blood pressure.

## Conclusions

Our study of lowland Nepalese children indicates that components of growth in early life contribute to the “metabolic capacity” to regulate blood pressure, and that adiposity is the main somatic component of “metabolic load” that challenges this aspect of homeostasis. Maternal capital shapes both metabolic capacity and metabolic load in the offspring. Further follow-up will be required to determine whether the existing physiological variability shapes health over the longer term.

## Figures and Tables

**Table 1:** Description of the sample stratified by gender

	Girls			Boys		
	*n*	Mean	SD	*n*	Mean	SD
**Offspring trait at birth**						
Weight (kg)	405	2.73*	0.40	434	2.82	0.43
Length (cm)	405	48.6*	2.9	430	49.1	2.9
**Offspring trait at 2 years**						
Weight (kg)	405	10.4*	1.3	436	11.1	1.5
Height (cm)	405	82.7*	4.4	436	84.7	4.8
BMI (kg/m^2^)	405	15.1*	1.3	436	15.5	1.5
**Offspring trait at 8 years**						
Weight (kg)	405	19.6*	3.0	436	20.5	3.5
Height (cm)	405	120.2*	5.9	436	121.2	6.0
BMI (kg/m^2^)	405	13.5*	1.2	436	13.9	1.4
Sitting height (cm)	405	63.8*	2.9	436	64.5	3.0
Leg length (cm)	405	56.4	3.6	436	56.7	3.5
Relative leg length	404	0.469*	0.010	436	0.468	0.010
Lean mass (kg)	404	16.5*	2.2	436	17.5	2.5
Fat mass (kg)	404	3.15*	1.32	436	3.04	1.62
Sum of skinfolds (mm)	405	22.2*	6.4	435	20.0	7.3
Systolic blood pressure (mm Hg)	404	98.1	7.1	436	98.0	7.7
Diastolic blood pressure (mm Hg)	404	61.4	7.1	436	60.9	8.1
Kidney length (cm)	404	8.02	0.53	435	8.08	0.55
Kidney AP diameter (cm)	403	3.10*	0.24	434	3.17	0.25
Kidney area (cm^2^)	404	78.6	9.2	434	80.9	10.2
Maternal trait						
Height (cm)	404	150.7	5.5	434	150.8	5.2
BMI (kg/m^2^)	404	19.6	2.1	434	19.7	2.3

* Significantly different from boys *P* < 0.05.

**Table 2:** Correlations of size at birth or maternal height with later body size, body composition and blood pressure

	Birth weight		Birth length		Maternal height		Maternal BMI	
	*r*	*P*	*r*	*P*	*r*	*P*	*r*	*P*
Weight	0.31	<0.0001	0.25	<0.0001	0.29	<0.0001	0.24	<0.0001
Height	0.24	<0.0001	0.24	<0.0001	0.35	<0.0001	0.13	0.0002
BMI	0.26	<0.0001	0.14	0.0016	0.08	0.0004	0.27	<0.0001
Sitting height	0.27	<0.0001	0.20	<0.0001	0.34	<0.0001	0.15	<0.0001
Leg length	0.20	<0.0001	0.19	<0.0001	0.32	<0.0001	0.05	ns
Relative leg length	0.01	ns	0.03	ns	0.13	0.012	–0.08	ns
Lean mass	0.30	<0.0001	0.24	<0.0001	0.29	<0.0001	0.20	<0.0001
Head girth	0.30	<0.0001	0.21	<0.0001	0.19	<0.0001	0.12	0.002
Ln Fat mass	0.15	<0.0001	0.13	0.0023	0.15	0.03	0.18	<0.0001
Ln Sum skinfolds	0.10	0.0004	0.03	ns	0.04	ns	0.17	<0.0001
Systolic blood pressure	–0.07	ns	–0.01	ns	0.03	ns	0.05	ns
Diastolic blood pressure	–0.05	ns	–0.00	ns	0.03	ns	0.05	ns
Kidney length	0.22	<0.0001	0.19	<0.0001	0.17	<0.0001	0.07	ns
Kidney AP diameter	0.17	<0.0001	0.12	0.0024	0.09	0.0005	0.05	ns
Kidney area	0.21	<0.0001	0.14	<0.0001	0.15	<0.0001	0.08	0.0075

**Table 3:** Correlations of kidney dimensions with body size and body composition at 8 years

	Kidney length		Kidney AP diameter		Kidney area	
	*r*	*P*	*r*	*P*	*r*	*P*
Weight	0.49	<0.0001	0.35	<0.0001	0.49	<0.0001
Height	0.50	<0.0001	0.30	<0.0001	0.49	<0.0001
BMI	0.25	<0.0001	0.27	<0.0001	0.32	<0.0001
Sitting height	0.53	<0.0001	0.30	<0.0001	0.47	<0.0001
Leg length	0.45	<0.0001	0.26	<0.0001	0.40	<0.0001
Relative leg length	0.05	0.2	0.06	0.07	0.06	0.059
Lean mass	0.47	<0.0001	0.33	<0.0001	0.49	<0.0001
Ln fat mass	0.26	<0.0001	0.23	<0.0001	0.29	<0.0001
Ln sum skinfolds	0.14	<0.0001	0.09	<0.0001	0.17	<0.0001
Systolic blood pressure	–0.01	0.7	0.09	0.006	0.05	0.0089
Diastolic blood pressure	–0.05	0.4	0.02	0.8	–0.02	0.8

AP, antero-posterior diameter.

**Table 4:** Regression statistics for the capacity-load model of blood pressure

	Systolic BP				Diastolic BP			
	*B*	SE	*P*	r^2^	*B*	SE	*P*	r^2^
Constant	98.19	1.78	<0.0001	0.038	61.44	1.82	<0.0001	0.035
Ln fat mass (kg)	3.417	0.611	<0.0001		3.368	0.626	<0.0001	
Birth weight (kg)	–1.316	0.628	0.036		–1.328	0.642	0.039	
Constant	91.22	3.18	<0.0001	0.016	53.55	3.02	<0.0001	0.008
Ln sum skinfolds (mm)	3.234	0.957	0.0008		2.553	1.002	0.011	
Birth weight	–1.057	0.616	0.086					
Constant	96.53	5.79	<0.0001	0.040	70.63	6.79	<0.0001	0.038
Ln fat mass (kg)	3.653	0.659	<0.0001		4.839	0.718	<0.0001	
Sitting height (cm)	0.224	0.121	0.065		0.030	0.142	0.8	
Leg length (cm)	–0.294	0.103	0.004		–0.297	0.120	0.013	
Constant	132.61	11.36	<0.0001	0.044	77.87	11.72	<0.0001	0.035
Ln fat mass (kg)	3.438	0.603	<0.0001		3.376	0.622	<0.0001	
Relative leg length	–81.415	24.41	0.0009		–43.064	25.20	0.088	
Constant	98.43	5.94	<0.0001	0.043	71.94	4.54	<0.0001	0.042
Ln fat (kg)	3.824	0.677	<0.0001		4.130	0.680	<0.0001	
Birth weight (kg)	–1.368	0.642	0.033		–1.173	0.001	0.068	
Sitting height (cm)	0.269	0.126	0.033					
Leg length (cm)	–0.315	0.105	0.003		–0.207	0.082	0.013	

**Table 5:** Regression statistics for the capacity-load model of systolic blood pressure stratified by maternal trial allocation group

	Multiple micronutrient group				Iron/folic acid group			
	*B*	SE	*p*	r^2^	*B*	SE	^*p*^	r^2^
Constant	131.67	16.92	<0.0001	0.044	137.46	18.08	<0.0001	0.072
Ln Fat (kg)	4.877	0.998	<0.0001		2.821	0.863	0.001	
Birth weight (kg)	–0.002	0.001	0.048		–0.001	0.001	0.3	
Relative leg length	–69.50	35.58	0.052		–86.51	38.76	0.026	

**Table 6:** Regression statistics for the capacity-load model of blood pressure incorporating kidney measurements

	Systolic BP				Diastolic BP			
Predictor	*B*	SE	*P*-value	_*r*_ **2**	*B*	SE	*P*-value	*r* ^2^
Constant	137.43	11.86	<0.0001	0.045	84.34	11.91	<0.0001	0.040
Ln fat mass	3.581	0.647	<0.0001		3.880	0.650	<0.0001	
Birth weight	–1.381	0.639	0.031		–1.232	0.642	0.055	
Relative leg length	–86.725	25.03	0.0006		–42.522	25.140	0.091	
Kidney area	0.018	0.028	0.5		–0.048	0.028	–0.089	
Constant	139.61	11.98	<0.0001	0.061	90.48	12.55	<0.0001	0.044
Ln fat mass (kg)	3.732	0.629	<0.0001		4.004	0.654	<0.0001	
Birth weight (kg)	–1.394	0.617	0.024		–1.200	0.637	0.060	
Relative leg length	–86.11	24.23	0.0004		–43.37	25.11	0.084	
Kidney length (cm)	–1.383	0.529	0.0091		–1.142	0.515	0.024	
Kidney AP diameter (cm)	3.161	1.132	0.0054					
Constant	96.02	6.18	<0.0001	0.053				
Ln fat mass (kg)	3.758	0.678	<0.0001					
Birth weight (kg)	–1.472	0.643	0.022					
Sitting height (cm)	0.322	0.132	0.015					
Leg length (cm)	–0.318	0.105	0.003					
Kidney length (cm)	–1.268	0.595	0.033					
Kidney AP diameter (cm)	3.117	1.168	0.008					
Constant	120.61	12.00	<0.0001	0.037				
Ln sum skinfolds (mm)	3.267	0.962	0.0007					
Birth weight (kg)	–1.134	0.625	0.070					
Relative leg length	–69.22	24.35	0.0046					
Kidney length (cm)	–1.038	0.532	0.0013					
Kidney AP diameter (cm)	3.267	0.962	0.0007					

BP, blood pressure; AP, antero-posterior diameter.
